# Microbial profile and putative microbial origin of anorectal abscess based on 16S rDNA sequencing

**DOI:** 10.3389/fcimb.2025.1587862

**Published:** 2025-10-30

**Authors:** Shanzhong Chen, Chun Dai, Hongyuan Zhao, Yifan Gu, Kuijun Sun, Xingran Yao, Feng Jiang, Renjie Shi

**Affiliations:** ^1^ Department of Anorectal Surgery, People’s Hospital of Yangzhong, Zhenjiang, Jiangsu, China; ^2^ First Clinical Medical College, Nanjing University of Chinese Medicine, Nanjing, Jiangsu, China; ^3^ Department of Gastrointestinal Surgery, People’s Hospital of Yangzhong, Zhenjiang, Jiangsu, China; ^4^ Department of Colorectal Surgery, The Affiliated Hospital of Nanjing University of Chinese Medicine, Nanjing, Jiangsu, China

**Keywords:** anorectal abscess, microbial profile, microbial origin, gut microbiota, 16s rDNA sequencing

## Abstract

**Background:**

Anorectal abscess is a common bacterial infectious disease; however, its pathogenesis remains unclear. Although there is emerging evidence linking gut microbiota to anorectal abscesses, knowledge of the microbial origin of this disease remains limited. This study analyzed the microbial profile of anorectal abscesses using 16S rDNA sequencing and investigated their microbial origin with the aim of providing a reference for subsequent studies.

**Methods:**

We recruited 60 patients with anorectal abscesses (AA group) and 26 healthy individuals (HC group) and used 16S rDNA V3-V4 hypervariable region gene sequencing to analyze the microbiota in feces, pus, and perianal buttock skin samples. The gut microbiota and perianal buttock skin flora were compared between the two groups, pus flora was analyzed in relation to the gut and perianal buttock skin microbiota, and microbial traceability analysis of pus was performed.

**Results:**

There were significant differences in the gut microbiota between the AA and HC groups. *Escherichia-Shigella* and *Prevotella* were significantly more prevalent, and *Bacteroides*, *Faecalibacterium*, and *Blautia* were decreased at the genus level in the AA group. *Escherichia-Shigella* and *Prevotella* were the main differential bacteria in the AA group and could be considered signature microbes of anorectal abscess. Kyoto Encyclopedia of Genes and Genomes (KEGG) analysis also showed significant differences between the two groups, with the AA group showing the most enrichment for protein families: metabolism. No significant difference in the perianal buttock skin flora was found between the two groups. The gut microbiota of patients with anorectal abscesses is similar to the pus flora than perianal buttock skin flora; hence, the gut microbiota is the putative microbial origin of the pus flora.

**Conclusion:**

Our study provides a microbiomic panorama and putative microbial origin of anorectal abscesses, potentially offering new insights into their etiology, prevention, and treatment.

## 1 Introduction

An anorectal abscess is a common surgical condition that presents as a collection of pus in the soft tissues and interstitial spaces around the anorectum, leading to symptoms including redness, swelling, and pain, and in severe cases, progressing to life-threatening necrotizing fasciitis (Lohsiriwat, 2016). The vast majority of anorectal abscesses are thought to be caused by cryptoglandular infections, which can spread in multiple directions and lead to different types of abscesses (Pearce et al., 2016), but their pathogenesis remains unclear. The cryptoglandular infection doctrine is currently the most widely accepted theory of the etiology of anorectal abscesses, first proposed by Eisenhammer in 1956 and further supported by anatomical evidence from Parks in 1961 (Parks, 1961). It has been speculated that the cause of anal gland infection is obstruction of the anal gland outlet duct by fecal material or trauma, causing the secretions in the gland ducts to not drain properly into the anal canal, leading to infection and pus. However, there is insufficient evidence to support this hypothesis (Gosselink et al., 2015). Once the disease is clearly diagnosed, surgical treatment is preferred. However, recurrence, anal fistula formation, slow wound healing, and other poor outcomes are common, imposing a heavy burden on both surgeons and patients (Sahnan et al., 2017). It is also worth noting that many anorectal abscesses heal completely without recurrence after simple incision and drainage (Hamadani et al., 2009). Previous studies on anorectal abscesses have focused on precise preoperative anatomical assessment and improvement of surgical approaches, while neglecting etiological studies, which may explain the lack of success in improving postoperative prognosis despite the proliferation of new surgical techniques. Therefore, there is an urgent need to develop new strategies to prevent and treat this disease.

The microbiome has a profound impact on human health and disease (Turnbaugh et al., 2007). The microbial communities of each organ are different, indicating that their effects on inflammation are likely to be organ-specific (Schwabe and Jobin, 2013). The gut microbiome is one of the most important microbiomes and is also a large ecosystem, more than 99% of which consists of bacteria involved in a wide range of physiological and pathological activities (Lv et al., 2023). As a purulent disease caused by bacterial infection, the etiology, development, and prognosis of anorectal abscesses are closely related to pathogenic bacteria, especially enterogenic microorganisms (Grace et al., 1982). Our group recently found that chronic diarrhea associated with intestinal dysbiosis is an independent risk factor for the recurrence of anorectal abscesses, and that patients with pus cultures of *Escherichia coli* and *Klebsiella pneumoniae* had a higher likelihood of recurrence (Chen et al., 2024). Microbiological research can provide insights into the clinical challenges of anorectal abscess (Sahnan et al., 2019).

Controversy remains over whether pus from patients with anorectal abscess should be routinely cultured. Proponents argue that the culture of pathogenic microorganisms is necessary not only to predict the likelihood of fistula formation after anorectal abscess surgery but also to guide the precise use of antibiotics and prevent antibiotic resistance (Eykyn and Grace, 1986; Bender et al., 2022; Guner Ozenen et al., 2023). Opponents argue that this step is unnecessary as it has no impact on clinical management, patient prognosis, or recurrence prediction (Xu et al., 2016; Seow-En and Ngu, 2017; Lalou et al., 2020). In addition, previous studies have used traditional culture methods and focused only on pus, making the results less precise and comprehensive. The advent of high-throughput 16S rDNA sequencing technology has provided a powerful tool for a more comprehensive analysis of the composition and function of microbial communities. The application of this technology in the study of anorectal abscesses can facilitate the elucidation of their etiology. A previous study used 16S rRNA gene sequencing to detect and compare the gut microbiota of healthy individuals and patients with perianal abscess, and found significant differences in both flora abundance and diversity between the groups (Yin et al., 2023). However, it was difficult to comprehensively characterize the microorganisms in anorectal abscesses and infer the source of infection because of the lack of paired pus and perianal skin samples. Another previous study conducted 16S rRNA gene sequencing on samples of anal skin, feces and pus from patients with perianal abscesses. The results indicated that the bacterial composition of the pus may originate from the skin and feces, as well as potentially other sources (Han et al., 2025). However, due to the absence of healthy control samples and microbial traceability analysis, the origin of the pus microorganisms remains unclear. Bayesian source tracking is used to determine the origins of bacterial contaminants and other microorganisms. This method uses 16S rRNA gene sequencing to quantify the proportional contributions of multiple potential source environments to a specific sink environment. Since its introduction, SourceTracker has been widely applied in various fields, ranging from environmental contamination studies to research on human behaviors. To date, the method has been cited over 1,400 times and is widely recognized as an effective tool for predicting microbial sources (Knights et al., 2011; McGhee et al., 2020; Fang et al., 2025).

The lack of a systematic comparison of microbial differences and source-tracking analysis of anorectal abscesses provided the rationale for conducting this study. We hypothesized that patients with anorectal abscesses have a different gut microbiota but similar perianal buttock skin flora to that in healthy individuals. We further investigated the relationship between the gut, pus, and perianal buttock skin microbiota in patients with anorectal abscesses to assess the potential role of gut microbiota in the pathogenesis, development, and prognosis of anorectal abscesses.

## 2 Materials and methods

### 2.1 Study population and protocol

We recruited 60 consecutive patients with anorectal abscesses (AA group) who underwent surgical treatment at the Department of Anorectal Surgery of Yangzhong People’s Hospital between October 2023 and June 2024, and 26 healthy individuals of matched sex and age who were confirmed by the hospital’s Health Screening Center during the same period to serve as a healthy control group (HC group). The study was conducted in accordance with the Declaration of Helsinki and all participants provided written informed consent. This study was approved by the Ethics Committee of Yangzhong People’s Hospital (No. KY202309).

Patient inclusion criteria were as follows: 1) diagnosed with anorectal abscess and operated on in our hospital; 2) aged 18–70 years, male or female; 3) no serious underlying diseases or contraindications to surgery; and 4) voluntary participation in this study and signing of an informed consent form.

The exclusion criteria were as follows: 1) non-cryptoglandular anorectal abscesses (e.g., due to trauma); 2) those who had used antibiotics, probiotics, or drugs affecting the gut microbiota within 3 months prior to enrolment; 3) those with inflammatory bowel disease, malignancy, tuberculosis, or human immunodeficiency virus; 4) skin diseases or infection of the buttock; and 5) pregnant or in the perinatal period.

### 2.2 Sample collection

**Figure 1:**
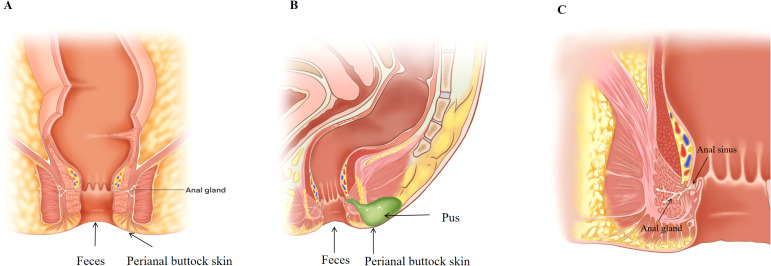
**(A)** Healthy control group sampling sites. **(B)** Anorectal abscess group sampling sites. **(C)** Schematic diagram of the anal gland.

Three samples were collected from each patient and two from healthy individuals, as shown in **Figures 1A, B**. Freshly collected fecal samples (≥0.5 g) from the HC and AA groups (preoperative) were placed in sterile tubes. Samples of pus from the AA group were collected during surgery. Prior to collection, the skin was disinfected and ≥1 ml of pus was aspirated using a sterile syringe and placed in a sterile tubes. This prevented cross-contamination of the samples. For perianal buttock skin sample collection, following a previously described method (Cui et al., 2023), participants were instructed not to wash their perianal buttock skin for 24 h prior to sampling. Sterile cotton swabs of Tris-EDTA and 0.5% Tween 20 solution were used to repeatedly wipe an area of approximately 4 × 4 cm on the perianal buttock skin for approximately 30 s with appropriate pressure, and then the tip of the swab was cut off with sterile medical scissors and placed in a sterile tube. All samples were stored at -80°C within 15 minutes of collection until further processing.

### 2.3 DNA extraction and 16S rDNA amplification sequencing

Total genomic DNA was extracted using the cetyltrimethylammonium bromide (CTAB) method. The hypervariable V3-V4 regions of bacterial 16S rDNA genes were amplified by polymerase chain reaction (PCR) using barcoded universal primers 341F (5′-CCTAYGGGRBGCASCAG-3′) and 806R (5′-GGACTACNNGGGTATCTAAT-3′). PCR amplicons were pooled in equimolar ratios and purified using the QIAquick Gel Extraction Kit (Qiagen, Hilden, Germany). Sequencing libraries were prepared using the TruSeq^®^ DNA PCR-Free Library Preparation Kit (Illumina, San Diego, CA, USA) according to the manufacturer’s protocols, incorporating unique dual-index adapters for sample multiplexing. Paired-end sequencing (2 × 250 bp) was subsequently performed on an Illumina NovaSeq 6000 platform (Nanjing Fengzi Bio-Pharm Technology Co., Ltd., Nanjing, China).

### 2.4 Data analysis

Raw paired-end FASTQ data were initially processed with fastp using default parameters to remove low-quality sequences and adapter contaminants, after which reads were merged into raw tags using VSEARCH (v2.18.0). The merged sequences underwent length filtering (retaining sequences between 200–550 bp) and quality control in MOTHUR, including removal of sequences containing homopolymers longer than 10 bp. In this sequencing, the mean sequencing depth was 100,796 tags and the cutoff for rarefaction was 21,599. Subsequent denoising was performed via the UNOISE3 algorithm in USEARCH with the minsize parameter set to 12, generating high-resolution amplicon sequence variants (ASVs). Taxonomic classification of representative ASVs was conducted using BLASTn alignment against the SILVA 138 SSU rRNA database with a 97% similarity threshold. The α-diversity metrics were computed using the vegan package (v2.6-4) and visualized using ggplot2 (v3.4.2) in R. β-diversity patterns were investigated using principal component analysis (PCA) and hierarchical clustering of ASVs profiles using the phyloseq package (v1.44.0). Principal coordinates analysis (PCoA) based on Bray-Curtis dissimilarity matrices was also employed to assess β-diversity differences in microbial community structure between sample groups, applying Cailliez correction to handle negative eigenvalues and ensure analytical accuracy. Intergroup differences in β-diversity were further assessed using permutational multivariate analysis of variance (PERMANOVA). Linear discriminant analysis effect size (LEfSe) analysis was performed using the microeco package (v0.11.0) to identify differentially abundant taxa. Metagenomic functional profiles were predicted using PICRUSt2 (v2.5.2) default with parameters. Diagnostic performance was assessed based on the receiver operating characteristic (ROC) curve using pROC analysis (v1.18.4). Microbial source traceability analysis was performed using the SourceTracker package in R according to a previous study (Knights et al., 2011). Differential abundance analysis was performed using the fitZig function from the *metagenomeSeq* package to implement the Metastats algorithm. Comparative metagenomic analysis was performed using STAMP (v2.1.3) with ggplot2-based visualization. All analyses were performed using the R statistical platform (v4.3.1), unless otherwise stated. Various statistical methods were used to evaluate differences between groups. Continuous data were assessed for normality using the Shapiro–Wilk test. Data that followed a normal distribution were compared between two groups using the independent samples t-test, with statistical significance set at P<0.05. For comparisons among multiple groups, one-way analysis of variance (ANOVA) was employed. If the ANOVA results indicated a statistically significant overall difference, *post-hoc* pairwise comparisons were conducted using the t-test with Bonferroni correction, where the significance level was adjusted to P< 0.05/n. For continuous data that did not follow a normal distribution, the Wilcoxon rank-sum test was used for comparisons between two groups, with a significance level of P<0.05. For comparisons among multiple groups, the Kruskal–Wallis test was applied. If the overall test result was statistically significant, *post-hoc* pairwise comparisons were performed using the Wilcoxon rank-sum test with Bonferroni correction, with the significance level set at P<0.05/n.

## 3 Results

### 3.1 Differences in gut microbiota between patients with anorectal abscess and healthy individuals

#### 3.1.1 Distinctions in the relative abundance of gut microbiota

**Figure 2:**
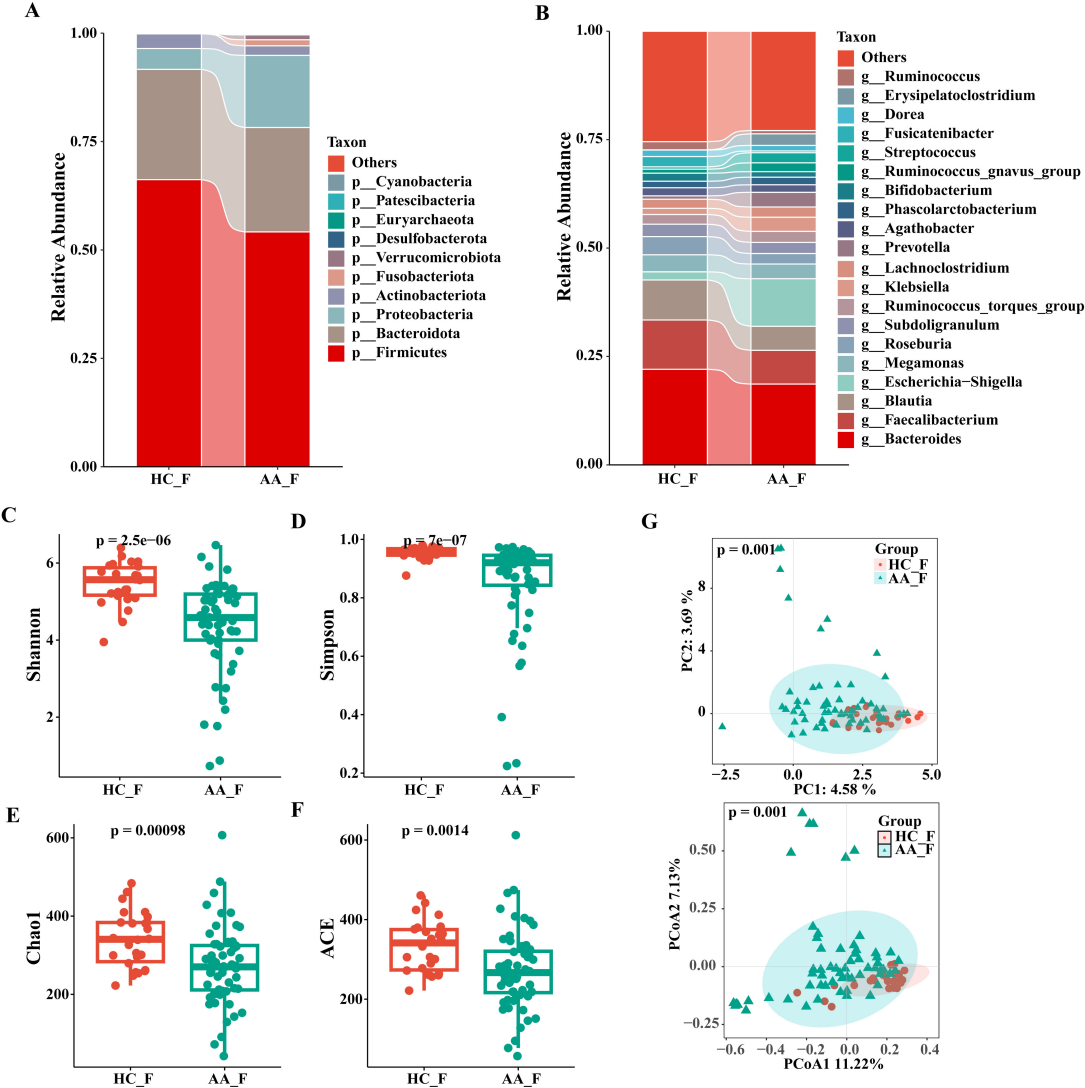
Composition and relative abundance of the gut microbiota at the phylum (top 10) and genus (top 20) level and analysis of α- and β-diversity in the HC and AA groups. **(A)** Phylum level. **(B)** Genus level. **(C)** Shannon index. **(D)** Simpson index. **(E)** Chao1 index. **(F)** ACE index. **(G)** Principal component analysis (PCA) and Principal coordinates analysis (PCoA). HC_F, gut microbiota in healthy control group. AA_F, gut microbiota in anorectal abscess group. **(C-F)** Wilcoxon rank-sum test showed that the differences in α-diversity among the two groups were significant (P< 0.001, Wilcoxon rank sum test), which meant that the species richness and evenness of gut microbiota in the HC group was higher than in the AA group. **(G)** Principal component analysis (PCA) and principal coordinate analysis (PCoA) by Bray-Curtis dissimilarity matrices (PERMANOVA, P = 0.001).

The composition and relative abundance of the gut microbiota at the phylum and genus levels in the two groups are shown in **Figures 2A, B**. At the phylum level, the top three phyla in both groups were *Firmicutes*, *Bacteroidota* and *Proteobacteria*. Compared to the HC group, the AA group had a higher relative abundance of *Proteobacteria* (16.6 vs. 4.8%, P<0.05), but a significantly lower abundance of *Firmicutes* (54.2 vs. 66.2%, P< 0.05) (**Supplementary Figure S1**). As shown in **Supplementary Figure S2**, the taxa were similar at the genus level, but the relative abundances varied considerably. *Faecalibacterium* (7.8 vs. 11.4%) and *Blautia* (5.5 vs. 9.2%) decreased significantly, whereas *Escherichia-Shigella* (11.0 vs. 1.9%) and *Prevotella* (3.4 vs. 0.8%) increased significantly in the AA group compared with that in the HC group (P<0.05). The relative abundance of *Bacteroides* in the AA group also showed a non-significant decrease compared with that in the HC group (18.6 vs. 22.0%). These data revealed that the microbial composition was similar but the relative abundance was distinct between the two groups.

#### 3.1.2 Differences in the α-diversity of gut microbiota

We used four indices of α-diversity to describe and compare the two groups, and the results showed that the Shannon, Simpson, Chao1, and ACE indices showed significant differences between the two groups and that the HC group had higher community richness and evenness than the AA group based on the Wilcoxon rank-sum test (P<0.001) (**Figures 2C–F**).

#### 3.1.3 Differences in the β-diversity of gut microbiota

Analysis of differences in the composition and distribution of gut microbiota between the two groups was performed using PCA and PCoA to demonstrate the β-diversity, which showed a significant difference between the two groups (P=0.001, PERMANOVA), suggesting that the microbial structures of the two groups were significantly different. (**Figure 2G**).

#### 3.1.4 Differential analysis of gut microbiota between the two groups by LEfSe

**Figure 3:**
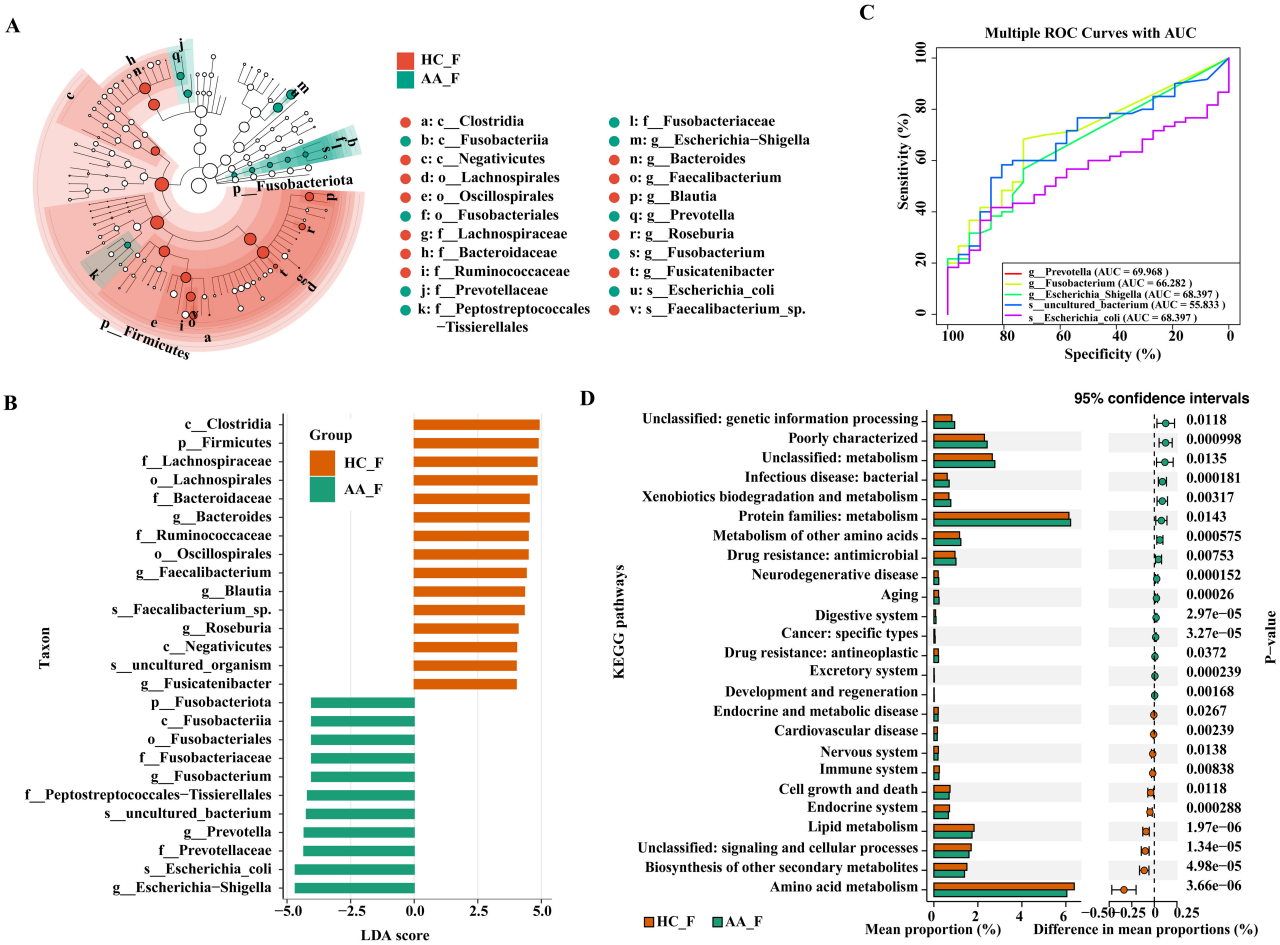
LEfSe, ROC, and KEGG analysis of the differential gut microbiota in the HC and AA groups. **(A)** Cladogram displaying the phylogenetic distribution of the microbiota, each circle represents a distinct phylogenetic hierarchy, progressing radially from phylum to genus from inner to outer rings. The diameter of each circle is proportional to the abundance of the corresponding taxon. **(B)** Histogram. The length of the columns indicates the value of the effect of the differential species. **(C)** ROC analysis. **(D)** KEGG pathway enrichment analysis (P< 0.05, Wilcoxon rank sum test). AUC, area under the curve. KEGG, Kyoto Encyclopedia of Genes and Genomes. LDA, linear discriminant analysis. LEfSe, linear discriminant analysis effect size. ROC, receiver operating characteristic curve. HC_F, gut microbiota in healthy control group. AA_F, gut microbiota in anorectal abscess group.

To further identify the core group of influential bacteria causing community differences, we performed a linear discriminant analysis (LDA) effect size analysis (LEfSe, LDA score >4, P< 0.05) to identify taxa with significant differences in abundance between the groups. Eight discriminating bacteria were found at the genus level, with three genera in the AA group and five in the HC group, respectively. In the AA group, in descending order of LDA value were *Escherichia−Shigella*, *Prevotella* and *Fusobacterium.* The genera *Bacteroides*, *Faecalibacterium*, *Blautia*, *Roseburia* and *Fusicatenibacter* were significantly more abundant in the HC group than in the AA group (**Figures 3A, B**).

#### 3.1.5 Screening for signature microbes of anorectal abscess

ROC curves were used to assess the sensitivity and accuracy of genus- and species-level bacteria with LDA > 4 in the AA group, as identified by LEfSe analysis. The area under the curve (AUC) of *Escherichia−Shigella* and *Prevotella* were 0.68397 and 0.69968, respectively, indicating that they are potential signature microbes for anorectal abscess (**Figure 3C**).

#### 3.1.6 Prediction of bacterial gene function

We employed PICRUSt2 for KEGG pathway enrichment analysis to predict gene function in intergroup bacteria. A total of 25 pathways were significantly different between the two groups, with 15 pathways enriched in the AA group and 10 pathways enriched in the HC group. The dominant functional enrichment of gut microbiota in the AA group was the protein family: metabolism, whereas amino acid metabolism was enriched in the HC group (P< 0.05, Wilcoxon rank sum test). Thus, the metabolic pathways of the gut microbiota differ between the two groups (**Figure 3D**).

### 3.2 Similarity of perianal buttock skin microbiota in patients with anorectal abscess and healthy individuals

#### 3.2.1 Similarity in composition and relative abundance of perianal buttock skin microbiota

**Figure 4:**
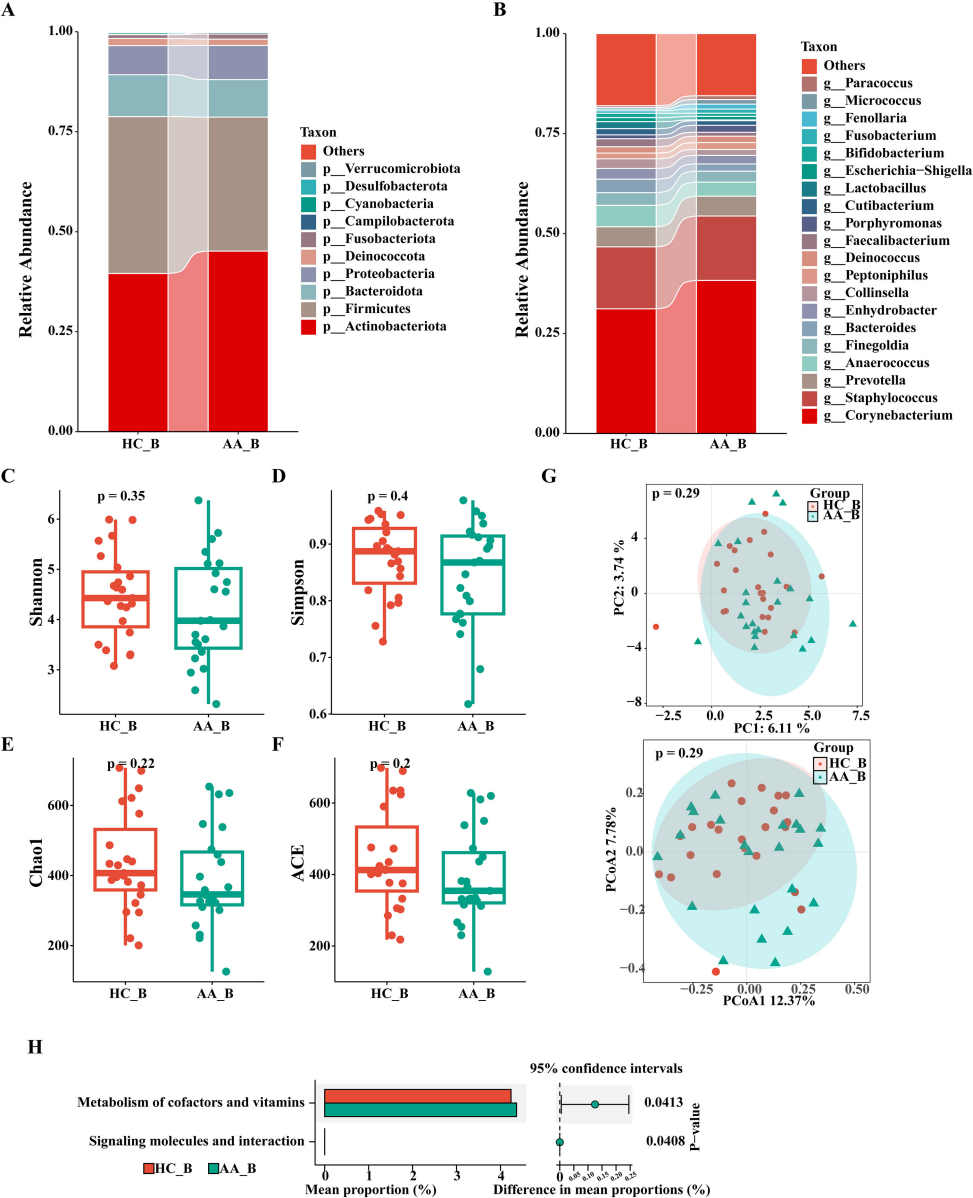
Analysis of the composition and gene function prediction of the perianal buttock skin microbiota in the HC_B and AA_B groups. **(A)** Phylum level. **(B)** Genus level. **(C)** Shannon index. **(D)** Simpson index. **(E)** Chao1 index. **(F)** ACE index. (P>0.05, Wilcoxon rank sum test). **(G)** Principal component analysis (PCA) and Principal coordinates analysis (PCoA) (P = 0.29, PERMANOVA). **(H)** KEGG analysis (P<0.05, Wilcoxon rank sum test). KEGG, Kyoto Encyclopedia of Genes and Genomes. HC_B, perianal buttock skin microbiota in the healthy control group. AA_B, perianal buttock skin microbiota in the anorectal abscess group.

As shown in **Figures 4A, B**, there were no significant differences in the composition and relative abundance of the perianal buttock skin microbiota between the two groups at the phylum and genus levels.

#### 3.2.2 No significant difference in the α- and β-diversity of perianal buttock skin microbiota

We used four indices of α-diversity to compare the two groups, which showed no significant differences in community richness and evenness between the two groups (P>0.05, Wilcoxon rank sum test) (**Figures 4C–F**). Furthermore, PCA and PCoA of the β-diversity showed overlap and a high degree of similarity between the two groups, suggesting that the microbiota structures of the two groups are similar (P=0.29, PERMANOVA) (**Figure 4G**).

#### 3.2.3 No significant difference in KEGG enrichment pathways

As shown in **Figure 4H**, the perianal buttock skin microbiota of the AA group was differentially enriched in only two metabolic pathways (P< 0.05, Wilcoxon rank sum test), with minimal differences compared with that in the HC group. Our result thus indicates that the metabolic pathways of the two groups are similar.

### 3.3 Putative microbial origin of anorectal abscess

#### 3.3.1 Composition and relative abundance of pus flora in the anorectal abscess resembles gut microbiota

**Figure 5:**
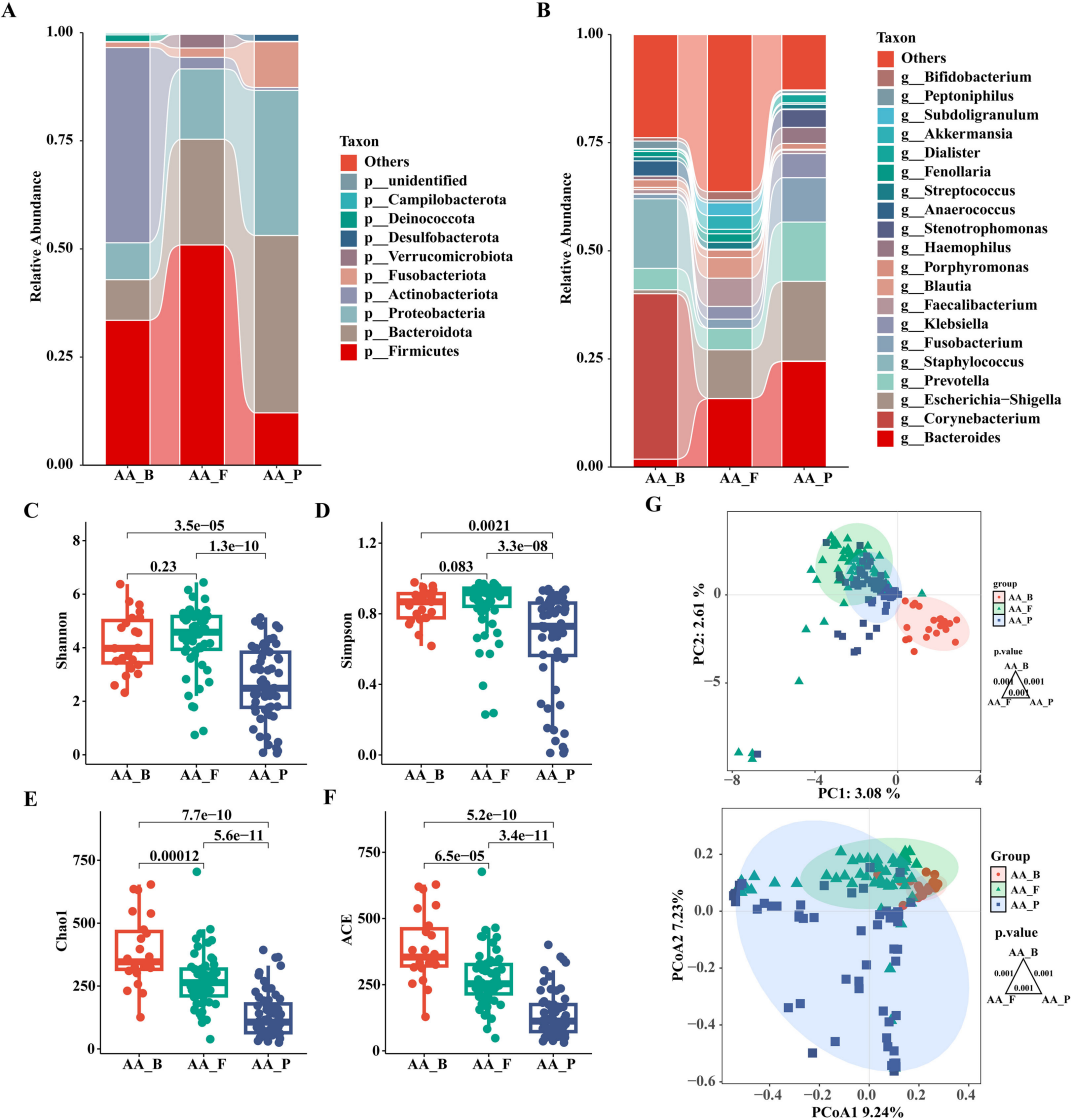
Analysis and comparison of the structures and communities of pus, gut microbiota, and perianal buttock skin flora in patients with anorectal abscess. **(A)** Phylum level. **(B)** Genus level. **(C)** Shannon index. **(D)** Simpson index. **(E)** Chao1 index. **(F)** ACE index. Overall differences in α diversity among the three groups were assessed using the Kruskal–Wallis test. For pairwise comparisons between groups, the Wilcoxon rank-sum test was employed, with Bonferroni correction applied to adjust the significance level (significance was defined as p<0.016). **(G)** Principal component analysis (PCA) and Principal coordinates analysis (PCoA). Each dot represents a sample, dots of the same color and shape are from the same group, and the closer the distance between two dots, the smaller the differences in community composition between them. Pairwise group differences were tested by PERMANOVA, P = 0.001, the Bonferroni-corrected significance threshold was set at P<0.016 (0.05/3 comparisons). AA_B, perianal buttock skin flora group. AA_F, gut microbiota group. AA_P, pus flora group.

We demonstrated intergroup relationships by comparing the composition and relative abundance of the pus flora, gut microbiota, and perianal buttock skin flora of patients with anorectal abscesses at the phylum and genus levels. At the phylum level, the top three in perianal buttock skin flora group (AA_B) were *Actinobacteriota* (45.1%), *Firmicutes* (33.5%), and *Bacteroidota* (9.4%); the top three of the pus group (AA_P) were *Bacteroidota* (41.0%), *Proteobacteria* (33.6%), and *Firmicutes* (12.1%); and the top three of the gut microbiota group (AA_F) were *Firmicutes* (50.9%), *Bacteroidota* (24.4%), and *Proteobacteria* (16.3%). At the genus level, the top three in the AA_B group were *Corynebacterium* (38.3%), *Staphylococcus* (16.1%), and *Prevotella* (5.0%); the top three in the AA_P group were *Bacteroides* (24. 4%), *Escherichia−Shigella* (18.4%), and *Prevotella* (13.7%); and the top three in the AA_F group were *Bacteroides* (15.8%), *Escherichia–Shigella* (11.3%), and *Faecalibacterium* (6.5%). These results show that the composition of pus flora in patients with anorectal abscesses more closely resembles that of the gut microbiota than that of the perianal buttock skin flora (**Figures 5A, B**). Although significant differences in relative bacterial abundance were observed between the AA_B, AA_P, and AA_F groups at the phylum and genus levels, these differences were smaller between the latter two groups. This suggests that the pus flora was more similar in composition and relative abundance to the gut microbiota than to the perianal buttock skin flora of patients with anorectal abscesses (**Supplementary Figures S3, S4**).

#### 3.3.2 *α*-diversity analysis in pus flora, gut microbiota, and perianal buttock skin flora of patients with anorectal abscesses

As shown in **Figures 5C–F**, the Shannon, Simpson, Chao1, and ACE α-diversity indices were compared between the three groups. No significant differences were found in the Shannon and Simpson indices between groups AA_B and AA_F (P> 0.016), whereas the remaining intergroup comparisons showed significant differences (P< 0.016). These results indicate that the bacterial communities of the perianal buttock skin flora and gut microbiota have similar community richness and evenness. However, the community richness and diversity of the pus flora are significantly lower.

#### 3.3.3 Composition and distribution of pus flora in anorectal abscesses is similar to that of the gut microbiota

PCA and PCoA were used to assess β-diversity between groups to demonstrate differences in flora composition and distribution. As shown in **Figure 5G**, although there was a significant difference among the three groups (P=0.001, PERMANOVA), groups AA_F and AA_P were closer together and partially overlapped, indicating that they were more similar than group AA_B in terms of community composition and distribution.

#### 3.3.4 Enriched KEGG pathways are similar in groups AA_F and AA_P compared to that in group AA_B

**Figure 6:**
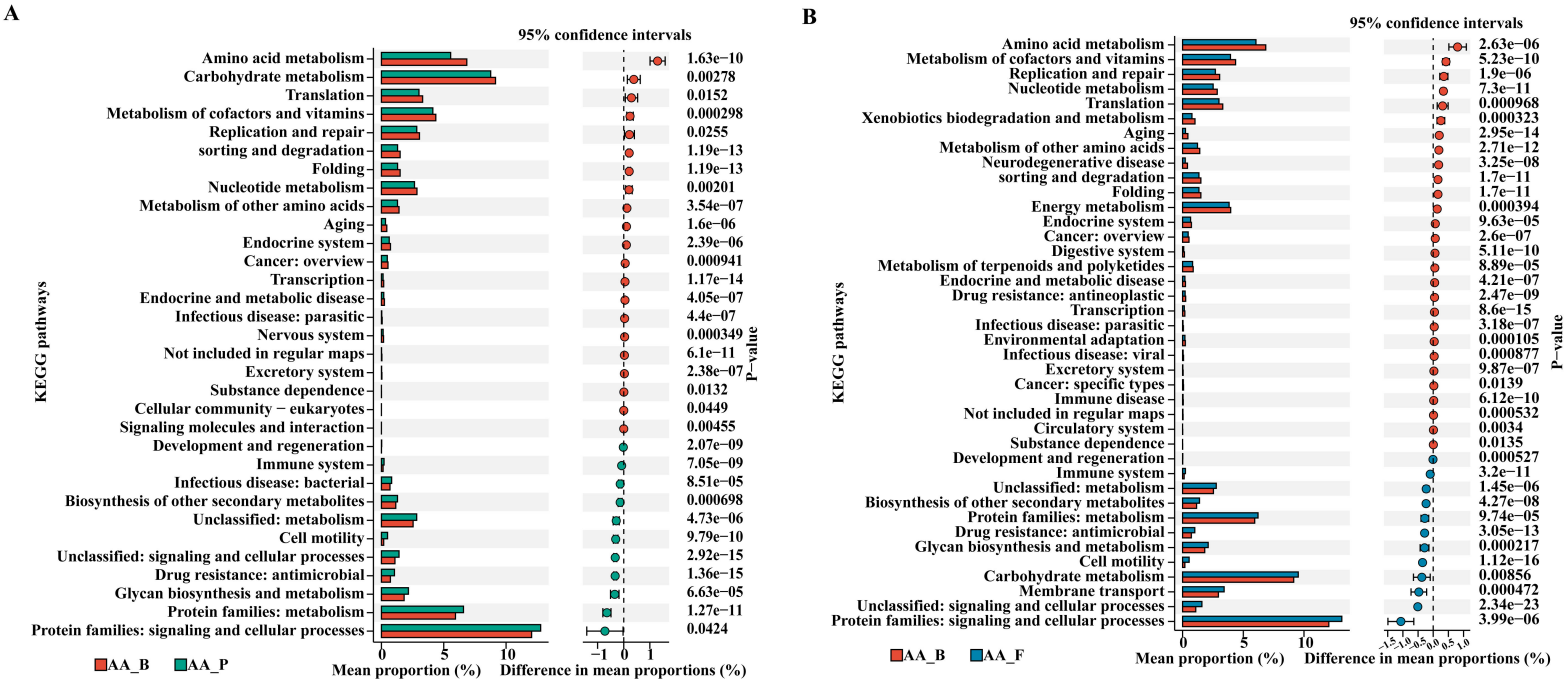
KEGG pathway enrichment analysis of AA_P and AA_F groups compared to AA_B group. **(A)** Between groups AA_B and AA_P. **(B)** Between groups AA_B and AA_F. KEGG, Kyoto Encyclopedia of Genes and Genomes. AA_B, perianal buttock skin flora group. AA_P, pus flora group. AA_F: gut microbiota group. (P< 0.05, Wilcoxon rank sum test).

As shown in **Figure 6**, by comparing the KEGG enrichment analysis with the AA_B group, we found that the differential metabolic pathways enriched in the AA_F and AA_P groups were similar, including protein families: signaling and cellular processes; protein families: metabolism, glycan biosynthesis, and metabolism; drug resistance: antimicrobial, immune system, cell motility, and biosynthesis of other secondary metabolites.

#### 3.3.5 Composition and structure of the pus flora in patients with perianal abscesses is more similar to their gut microbiota

**Figure 7:**
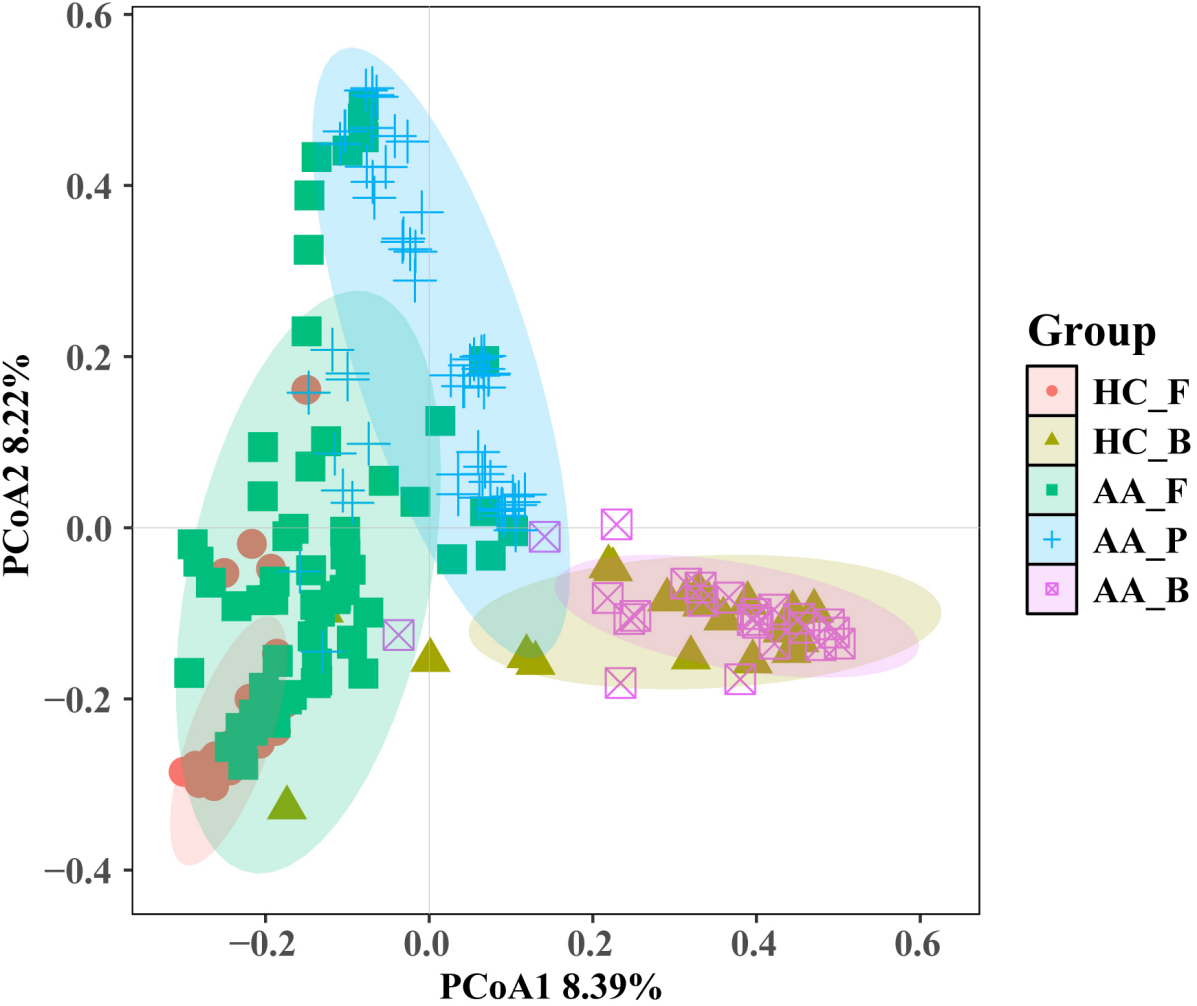
Principal coordinates analysis (PCoA) of microbial community composition across the five sample groups. HC_F, gut microbiota in the healthy control group. HC_B, perianal buttock skin flora in the healthy control group. AA_F, gut microbiota in the anorectal abscess group. AA_P, pus flora in the anorectal abscess group. AA_B, perianal buttock skin flora in the anorectal abscess group. Pairwise group differences were tested by PERMANOVA, the Bonferroni-corrected significance threshold was set at P<0.005 (0.05/10 comparisons). Pairwise comparisons showed significant differences (P=0.001) for all pairs except HC_B vs AA_B (P=0.29).

When all five sample groups were subjected to β-diversity analysis, results consistent with those presented in Section 3.3.3 were observed. This indicates that the pus flora composition and structure in patients with perianal abscesses is more similar to their gut microbiota than to that of other groups. As shown in **Figure 7**, microbial communities from the same sampling sites exhibited similarity (HC_F and AA_F; HC_B and AA_B). However, AA_P clustered most closely with AA_F, showing partial overlap, and was clearly separated from the other three groups.

#### 3.3.6 Microbial traceability analysis of pus

**Figure 8:**
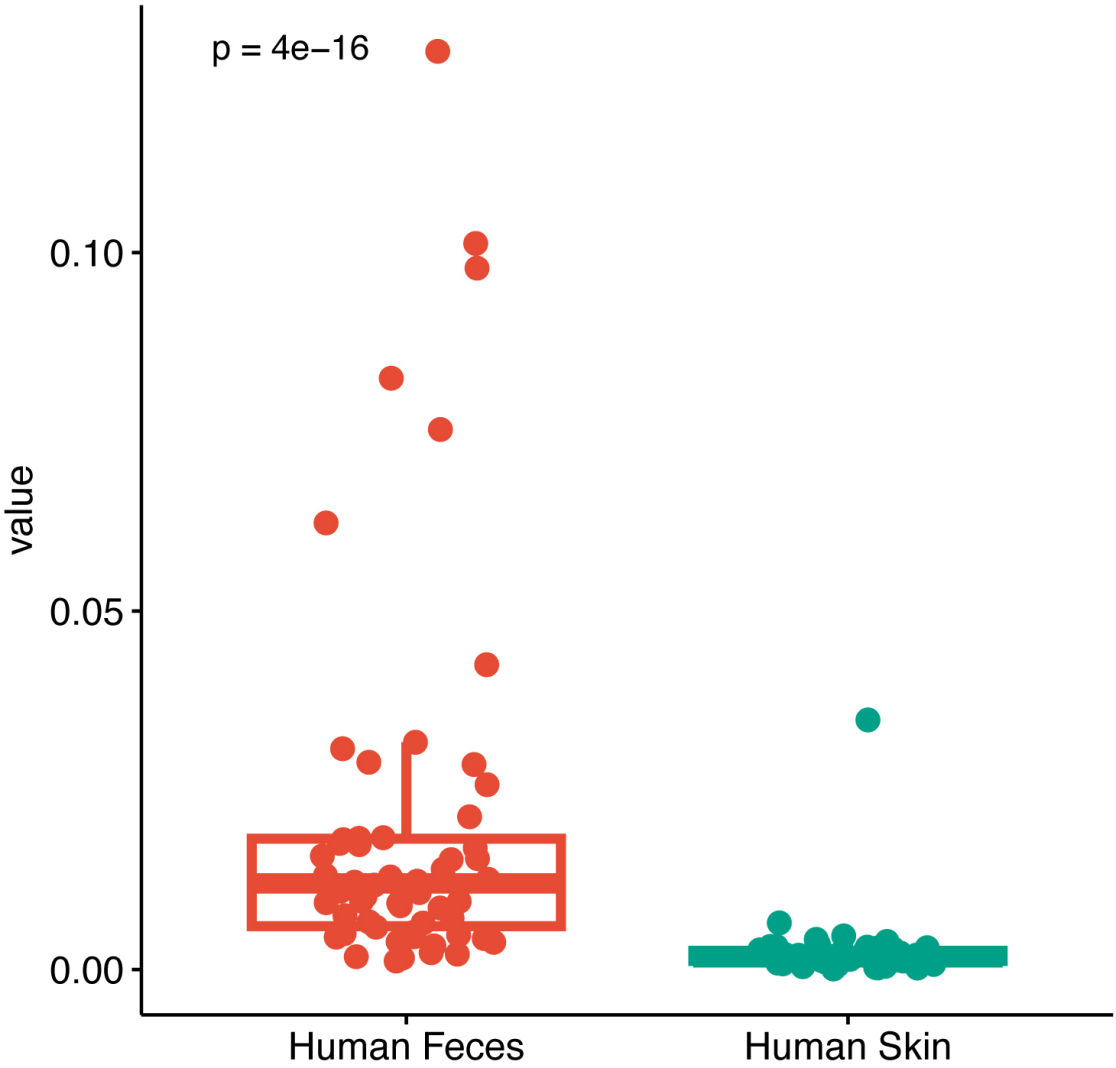
Microbial traceability analysis of pus. Fecal and perianal buttock skin samples were set as the source and pus as the target samples to be tested, and the contribution of the different source samples to the target samples was calculated to identify the microbial origins of the target samples (P< 0.001, Wilcoxon rank sum test).

Fecal and perianal buttock skin samples were set as the source and pus as the target samples to be tested, and the contribution of the different source samples to the target samples was calculated to identify the microbial origins of the target samples and their relationships. The results showed that the proportion of microbial communities of fecal origin in the patient’s pus was significantly higher than that of perianal buttock skin origin, suggesting that the gut microbiota is the potential microbial origin of the anorectal abscess and plays a role in its development and progression (P< 0.001, Wilcoxon rank sum test) (**Figure 8**).

## 4 Discussion

We studied the gut, pus, and perianal buttock skin microbiota of patients with anorectal abscesses, using fecal and perianal buttock skin samples from healthy individuals as controls. There were two main findings in our study. First, the gut microbiota of patients with anorectal abscess was significantly different from that of healthy individuals, but the perianal buttock skin flora was similar. Second, the gut microbiota is the putative microbial origin of anorectal abscesses, and gut dysbiosis may play an important role in the pathogenesis of anorectal abscesses. These data lay the foundation for exploring the role of gut dysbiosis in the pathogenesis, development, and prognosis of anorectal abscesses and provide new insights for the prevention and treatment of this disease.

The gut microbiota of patients with anorectal abscesses differs significantly from that of healthy individuals. The α-diversity analysis showed that there were significant differences in species richness and diversity between the two groups, and that the AA group was lower than the HC group, indicating that the gut microbiota of the AA group was in a relatively unhealthy state. Consistent with a previous study (Yin et al., 2023), β-diversity analysis showed significant differences in species composition and distribution despite partial overlap between the two groups, with greater differences between samples within the AA group. Although the microbiota composition was similar between the two groups at the phylum and genus level, there was a significant difference in the relative abundance of the flora between the groups, with a significant increase in the abundance of *Proteobacteria* at the phylum level, and with *Escherichia-Shigella* and *Prevotella* significantly enriched and *Bacteroides*, *Faecalibacterium* and *Blautia* decreased at the genus level in the AA group. Interestingly, this is consistent with previous studies on gut microbiota in patients with inflammatory bowel disease, which showed that reduced diversity, decreased *Firmicutes*, increased *Proteobacteria* (Matsuoka and Kanai, 2015), and anorectal abscess and anal fistula are common complications of inflammatory bowel disease. This phenomenon was also confirmed by LEfSe and ROC analysis. Healthy individuals have a low abundance of *Proteobacteria* in the gut; however, a significantly increased load is a marker of dysbiosis and disease (Shin et al., 2015). An increase in the abundance of *Escherichia-Shigella*, a common proinflammatory microbe, will disrupt intestinal homeostasis and put the gut into a pro-inflammatory state (Yang et al., 2022). *Prevotella* is considered a commensal bacterium, one of the three gut bacterial enterotypes; however, some *Prevotella* strains may be clinically important pathogens, and the mucosal inflammation mediated by them leads to the systemic dissemination of inflammatory mediators, bacteria, and bacterial products, which can participate in human diseases by promoting inflammation (Larsen, 2017). *Bacteroides* is an important cornerstone genus in the human gut that plays an important role in host immunity, nutrient metabolism, maintenance of intestinal homeostasis, and promotion of host health (Wang et al., 2021). *Faecalibacterium* has traditionally been considered a bioindicator of human health, with the inflammatory process becoming favorable when its population is decreased (Ferreira-Halder et al., 2017). *Blautia* is a potential probiotic genus that plays a role in maintaining the balance of the intestinal environment and preventing inflammation (Liu et al., 2021). KEGG analysis showed significant differences between the two groups, with the AA group being most enriched in protein families: metabolism, which can provide energy for biological activities, but can induce disease if excessively upregulated. The relationship between gut microbiota and perianal abscess has attracted growing interest in recent years. A recent study showed that patients with perianal abscesses have different gut microbiota, metabolites and metabolic pathways to healthy individuals. The species richness and diversity of the gut microbiota in patients are lower than in healthy individuals, indicating an unhealthy state. In the patient group, the abundance of the *Firmicutes* phylum decreased while that of the *Proteobacteria* phylum increased. The abundance of the *Faecalibacterium* and *Blautia* genera decreased while that of the *Escherichia-Shigella* genus increased (Ma et al., 2025). These results are consistent with our findings. Another study investigating the gut microbiota and relevant metabolites in infants with perianal abscesses revealed significant differences in the composition of gut microbiota and metabolites between healthy infants and those with abscesses, suggesting a potential link between the gut microbiota and perianal abscesses in infants (Wang et al., 2025). However, the microbial origin of perianal abscess—specifically, whether the microorganisms are derived from the gut or other sites—was not examined. Diabetes mellitus, obesity, smoking, alcohol intake, and diarrhea have been previously reported as risk factors for anorectal abscesses and fistulas (Wang et al., 2014; Chen et al., 2024), and these factors are often associated with intestinal flora dysbiosis (Cani, 2018; Redondo-Useros et al., 2020; Li et al., 2021); hence, it is reasonable to speculate that intestinal dysbiosis, characterized by increased harmful bacteria and decreased beneficial bacteria, is a possible etiology of anorectal abscesses. *Escherichia-Shigella* and *Prevotella* may serve as signature microbes or therapeutic targets to prevent postoperative recurrence of anorectal abscess and improve prognosis.

Multiple analysis of α-diversity, β-diversity, species composition and abundance, and KEGG pathways revealed no significant difference in the perianal buttock skin flora between patients with anorectal abscesses and healthy individuals. Anorectal abscess, a bacterial infectious disease, has no origin other than enteric or cutaneous. The former is usually presumed based on the doctrine of cryptoglandular infection, but this has not been supported by reliable evidence. To the best of our knowledge, this is the first study to compare the perianal buttock skin flora of patients with anorectal abscess and healthy individuals by gene sequencing, confirming our speculation that there was no significant difference between the two groups and that the patients’ perianal buttock skin flora did not show any dysbiosis. The above findings prompted us to further investigate the relationship between the gut, pus, and perianal buttock skin microbiota of patients with anorectal abscesses, and to explore the origin of the disease and possible mechanisms.

The gut microbiota of patients with anorectal abscesses is closer to the pus flora than to the perianal buttock skin flora. Although the pus flora was significantly different from both the gut microbiota and the perianal buttock skin flora in both the α- and β-diversity analyses, it was clear from the β-diversity analysis that the former two were close together and partially overlapped, whereas the latter was clearly isolated, a PCoA analysis of all five sample groups revealed similar trends, suggesting that the pus flora was more similar to the gut microbiota. This finding is further supported by the analysis of species composition and relative abundance: groups AA_P and AA_F differ in relative abundance, but the top three species at phylum level and the top two species at genus level are the same, and are clearly different from group AA_B. The two genera ranked second and third in the AA_P group, and *Escherichia-Shigella* and *Prevotella* are signature microbes of the group AA_F. A previous study has shown significant differences in bacterial composition between pus, anal skin, and feces. *Bacteroides* and *Escherichia-Shigella* dominate fecal samples, while *Staphylococcus* and *Corynebacterium* dominate anal skin samples (Han et al., 2025). These findings are consistent with our results. However, this study suggested that the microbes in perianal abscess may originate from the anal skin, feces, or other sources — a conclusion that differs from our findings. One possible reason for this discrepancy is that the aforementioned study did not use microbial source tracking analysis. Furthermore, the sampling site on the perianal skin used in that study may have been susceptible to fecal contamination. By contrast, our sampling was performed on the outer perianal skin of the buttocks, an area that is less likely to be affected by fecal exposure. This approach better preserves the integrity of the native perianal skin microbiota and provides a more accurate representation of its composition. In the KEGG enrichment analysis, with group AA_B as the control, it was found that groups AA_F and AA_P were differentially enriched in multiple identical metabolic pathways dominated by protein metabolism. This finding suggests that protein metabolism plays an important role in the progression of anorectal abscesses. Finally, we employed microbial traceability analysis to investigate the possible origin of the pus flora, and the results showed that the proportion of fecal origin was significantly higher than that of the perianal buttock skin, suggesting that the pus flora is more likely to originate from the gut microbiota than from the perianal buttock skin flora. Thus, the etiology of an anorectal abscess can be considered as a translocation of the gut microbiota. This result also raises the question: what is the mechanism by which the translocation of the gut flora leads to anorectal abscesses?

The pathogenesis of anorectal abscess may be because of intestinal flora dysbiosis resulting in mucin depletion in the anal glands, barrier disruption, and translocation of intestinal flora, and reversing this is key to preventing recurrence and anal fistula formation. As shown in **Figure 1C**, the anal glands are small glands that communicate with the anal canal through glandular ducts that open into the anal sinus and contain mucus-expressing MUC5AC, which was previously thought to be a simple lubricant that facilitates fecal elimination (Muranaka et al., 2018). Mucus is the main protective barrier against pathogens in the gastrointestinal tract and can be regulated by the microflora, and it contains water, immunoglobulins, and mucins (Pothuraju et al., 2020). Mucins are glycoproteins with a core backbone of proteins, and their glycosylation is essential for maintaining the mucosal barrier against infections (Linden et al., 2008). Mucin degradation is considered a primary step in the bacterial pathogenesis of many intestinal diseases. The absence of mucin-producing cells in the anal gland tissue of patients with anal fistulas (Mitalas et al., 2012) may explain the lack of spontaneous healing of anal fistulas. It is reasonable to speculate that mucus in the anal glands has an antimicrobial barrier function. Anorectal abscess and anal fistula are the acute and chronic phases of the same disease, but why do some anorectal abscesses heal after surgery while others form anal fistulas, and why does timely incision and drainage significantly reduce the incidence of anal fistula after abscess surgery (Yano et al., 2010; Chen et al., 2024)? One plausible explanation for this is that the degree of mucin depletion in the anal glands during the course of the disease is a determining factor. Gut dysbiosis, characterized by increased abundance of *Escherichia-Shigella* and *Prevotella*, may be responsible for the erosion of mucin in the anal glands. *Prevotella* is a mucin-degrading microbe that can destroy barrier integrity and trigger inflammation by over-degrading gastrointestinal mucin (Ley, 2016; Glover et al., 2022). This mechanism is supported by the KEGG analysis, which showed that the patients’ gut microbiota gene function was significantly enriched in protein family metabolism. If the anal gland barrier is disrupted, conditionally pathogenic bacteria (e.g., *Escherichia-Shigella*, *Bacteroides*, *Fusobacterium*, and *Klebsiella*) can migrate to the anorectal space and cause an anorectal abscess. It can be surmised that this trend can be reversed, as fistulas do not always form after simple incision and drainage of anorectal abscesses. If intestinal dysbiosis is corrected, and a prompt and adequate incision and drainage is performed, or other factors contributing to the destruction of the anal gland barrier are removed, and the anal gland structure is still intact, the damaged barrier has the potential to rebuild, and the anorectal abscess will not recur or form an anal fistula, which is supported by the results of our previous study (Chen et al., 2024).

Although this study provides a comprehensive landscape of the anorectal abscess microbiome and speculates on the microbial origin of anorectal abscesses, it has some limitations. First, a limitation of this study is that 16S rDNA sequencing makes it difficult to fully profile taxa below genus level, and providing relative abundance data rather than quantitative data (e.g., via qPCR). These constraints consequently hinder a comprehensive interpretation of the results. Second, fecal samples may not be fully representative of the anorectal microbiota. Third, the single-center nature of this study makes it susceptible to potential limitations in terms of generalizability.Fourth, this study provides ecological evidence supporting the gut as the potential microbial origin of anorectal abscesses. However, hypotheses regarding mucosal barrier breakdown and mucin depletion require further histological or animal model validation. Finally, as inflammation modulates the gut microbiota, it is important to consider that changes in the gut microbiota may be a consequence of the local or systemic inflammatory process triggered by an anorectal abscess rather than its cause. Targeted studies designed to trace the source of infection must be conducted to establish a more precise causal link.

Further studies are warranted to elucidate the mechanisms by which gut dysbiosis affects anorectal abscesses and the changes and differences in the gut microbiota between patients who develop anal fistulas after surgery and those who do not.

## 5 Conclusion

We provide a microbiomic panorama and a putative microbial origin of anorectal abscess. The gut microbiota of patients with anorectal abscess was different from that of healthy individuals, but the perianal buttock skin flora was similar, and the gut microbiota is the potential microbial origin of anorectal abscess. *Escherichia-Shigella* and *Prevotella* are signature microbes and potential therapeutic targets for anorectal abscess. Clinical trials based on gut microbiota modulation may be considered in the future for the prevention, treatment, and prognosis improvement of anorectal abscess.

## Data Availability

The datasets for this study can be found in the NCBI repository, accession number PRJNA1227892 (https://www.ncbi.nlm.nih.gov/sra/PRJNA1227892).
