# Supplementary material for: Microbial profile and putative microbial origin of anorectal abscess based on 16S rDNA sequencing
Source: Front Cell Infect Microbiol. 2025 Oct 30;15:1587862. doi: 10.3389/fcimb.2025.1587862 (PMC12611955; doi:10.3389/fcimb.2025.1587862)
Supplement: Supplementary file 1 [file DataSheet1.docx]

Supplementary Material

## Supplementary Figures


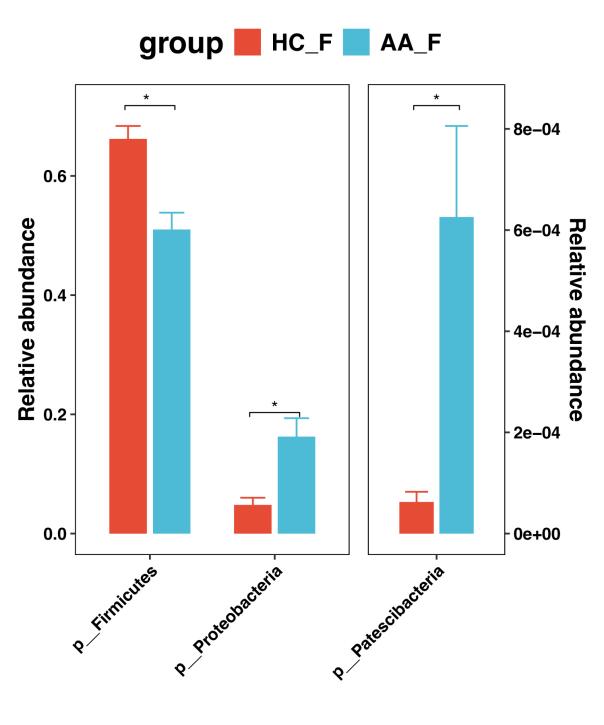


**Figure S1**

Relative bacterial abundance and differences at the phylum level in the HC and AA groups. The vertical axis is the proportion of phyla and the horizontal axis is the different bacteria phyla. HC_F, gut microbiota in healthy control group. AA_F, gut microbiota in anorectal abscess group. Differential abundance analysis of taxonomic groups at the phylum level between the HC and AA groups was performed by Metastats. (P-value < 0.05).


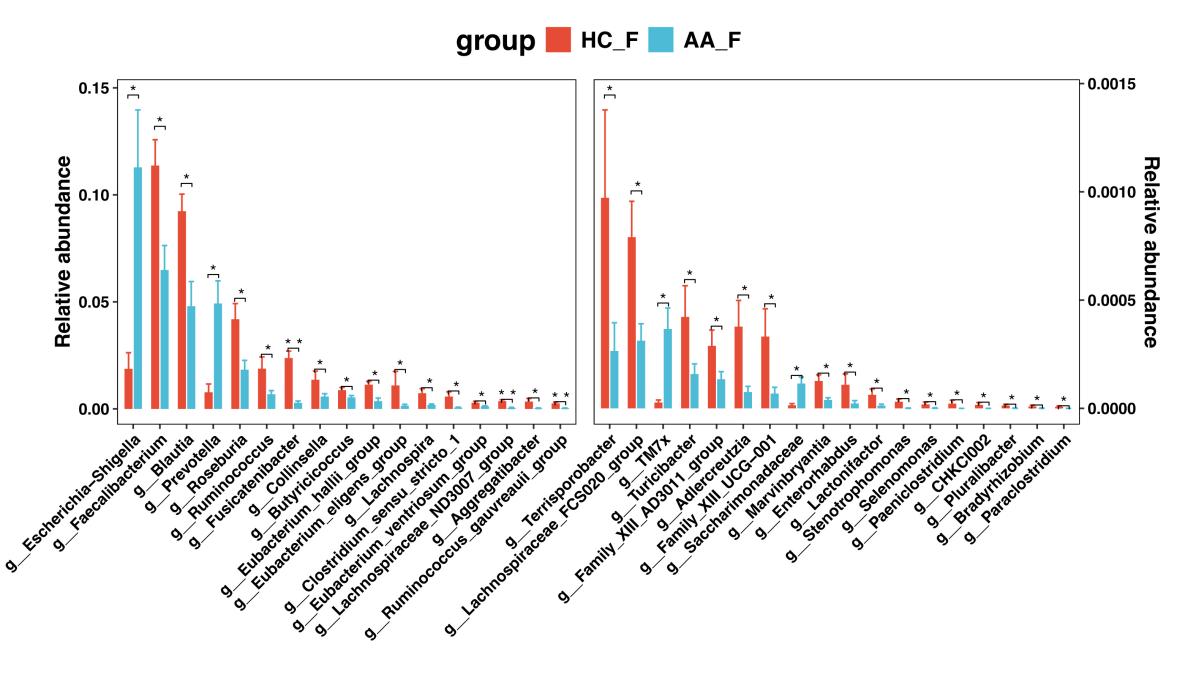


**Figure S2**

Relative bacterial abundance and differences at the genus level in the HC and AA groups. The vertical axis is the proportion of genera and the horizontal axis is the different bacteria genera. HC_F, gut microbiota in healthy control group. AA_F, gut microbiota in anorectal abscess group. Differential abundance analysis of taxonomic groups at the genus level between the HC and AA groups was performed by Metastats. (P-value < 0.05).


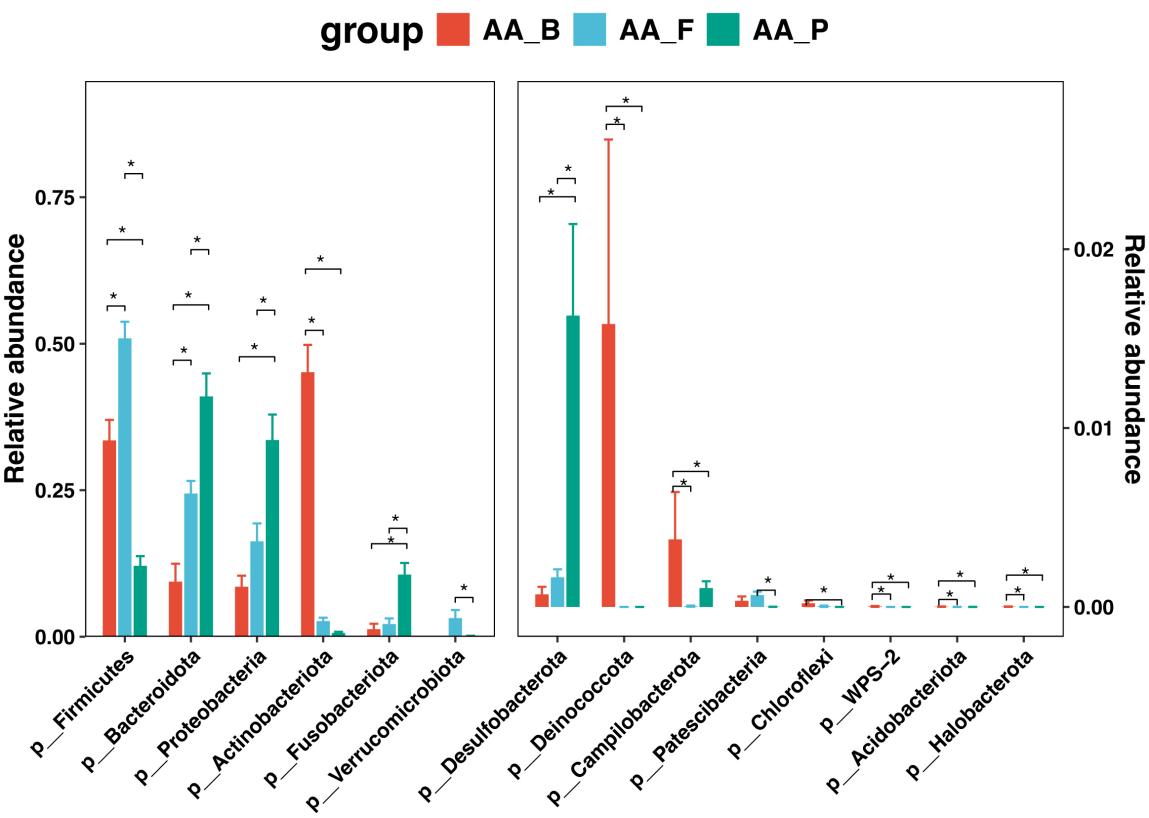


**Figure S3**

Relative bacterial abundance and differences at the phylum level in the AA_B, AA_F and AA_P groups. The vertical axis is the proportion of phyla and the horizontal axis is the different bacteria phyla. AA_B, perianal buttock skin flora group. AA_F, gut microbiota group. AA_P, pus flora group. Differential abundance analysis of taxonomic groups at the phylum level between the AA_B, AA_F and AA_P groups was performed by Metastats. (P-value < 0.05).


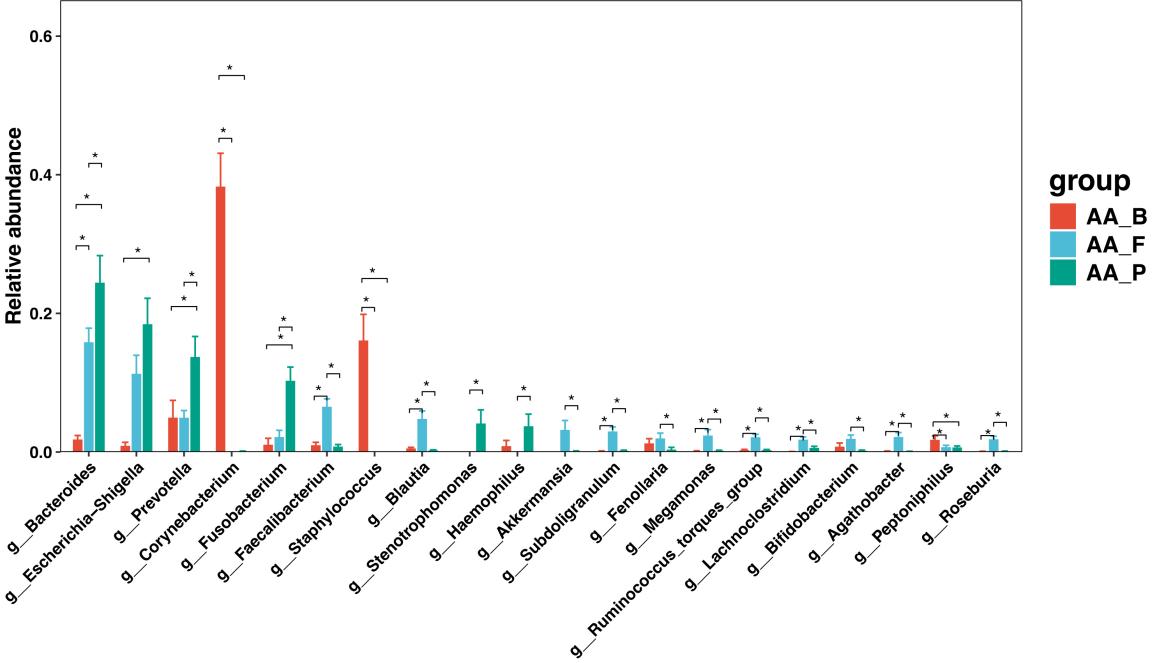


**Figure S4**

Relative bacterial abundance and differences at the genus level in the AA_B, AA_F and AA_P groups. The vertical axis is the proportion of genera and the horizontal axis is the different bacteria genera. AA_B, perianal buttock skin flora group. AA_F, gut microbiota group. AA_P, pus flora group. Differential abundance analysis of taxonomic groups at the genus level between the AA_B, AA_F and AA_P groups was performed by Metastats. (P-value < 0.05).
